# Retinal Microvascular Alterations in Hidradenitis Suppurativa Patients: A Pilot Study Using Optical Coherence Tomography Angiography

**DOI:** 10.3390/jcm13051464

**Published:** 2024-03-02

**Authors:** Marco Manfredini, Emanuele Ragusa, Matteo Gibertini, Laura Bigi, Barbara Ferrari, Claudia Lasagni, Cristina Magnoni, Andrea Lazzerini, Francesca Farnetani, Tommaso Verdina

**Affiliations:** 1 Dermatology Unit, Department of Surgical, Medical, Dental & Morphological Sciences with Interest Transplant, Oncological & Regenerative Medicine, University of Modena and Reggio Emilia, 41125 Modena, Italylasacla65@gmail.com (C.L.);; 2 Institute of Ophthalmology, Department of Surgical, Medical, Dental & Morphological Sciences with Interest Transplant, Oncological & Regenerative Medicine, University of Modena and Reggio Emilia, 41125 Modena, Italy

**Keywords:** hidradenitis suppurativa, optical coherence tomography angiography, retinal microvasculature, macula, optic nerve, vessel density

## Abstract

**Background**: Hidradenitis suppurativa (HS) is a relapsing–remitting inflammatory disease characterized by the progression of asymptomatic nodules to deep-seated lesions and fistula formation that leads to suppuration and scarring. Optical coherence tomography angiography (OCTA) is a new non-invasive imaging technique that carefully analyzes retinal microvasculature networks with high-resolution imaging. Recent studies have demonstrated that retinal vessel density and retinal perfusion reflect systemic inflammatory responses. This study’s aim was to analyze OCTA-derived retinal microvasculature parameters to understand if patients affected by HS and without any relevant ocular or systemic comorbidities showed impaired retinal vascular function and morphology. **Method**: We performed a case–control study of HS patients and age- and sex-matched control cohort. A total of 20 eyes from 10 HS patients and 30 eyes from 15 healthy controls were analyzed, and OCTA-derived microvasculature parameters were compared between groups. **Results**: OCTA images showed that HS patients, compared to healthy controls, were typically characterized by higher values of the foveal avascular zone (FAZ) both in the superficial capillary plexus (SCP) and in the deep capillary plexus (DCP), and by lower values of vessel density (VD)-SCP, VD-DCP, and vessel length density (VLD)-SCP in the foveal region. These findings partially reflect changes that have been demonstrated in diabetic patients that could be induced by a protracted metabolic or systemic inflammatory dysregulation. **Conclusions**: In conclusion, OCTA enables large-scale, non-invasive visual screening and follow-up of the retinal vasculature features, providing a new strategy for the prevention and monitoring of visual changes in HS patients.

## 1. Introduction

Hidradenitis suppurativa (HS) is a relapsing–remitting inflammatory disease characterized by the progression of asymptomatic nodules to deep-seated lesions and fistula formation. This leads to suppuration and scarring, with functional limitations and esthetic disadvantages [1,2]. The etiology of HS is multifactorial and involves both genetic and environmental factors leading to follicular occlusion, altered immunological response, and bacterial colonization [2,3,4]. Treatment commonly consists of topical and systemic therapies including antibiotics, anti-inflammatory drugs, retinoids, and more invasive surgical treatments, which must often be combined for the best outcomes [5,6,7,8]. Therapeutic approaches have evolved rapidly in the last decade and include the use of topical therapies, systemic antibiotics, hormonal therapies, and a wide range of immunomodulating medications [5].

Novel immunologic and large-scale proteomic studies of HS have demonstrated that the sera of patients with HS are characterized by a significant Th1/Th17 enrichment and upregulation of atherosclerosis-related proteins [9]. HS presents a greater serum inflammatory burden compared to psoriasis, even though several molecular and therapeutic similarities between the two diseases have been described [9,10,11]. In a recent study by Navrazhina et al., the proteins synergistically or additively induced by TNF and IL-17 were significantly augmented in both diseases, with HS presenting a greater enrichment of innate immunity response and positive regulation of immunoglobulin production pathways, with respect to psoriasis [9]. Persistent inflammation results in a large disease burden that encompasses not only the cutaneous manifestations but also other comorbidities and disease associations [2,12,13,14]. An association between HS and several other inflammatory and genetic disorders has also been reported in the literature, including acne conglobata, spondyloarthropathies, pyoderma gangrenosum, synovitis-acne-pustulosis-hyperostosis-osteitis syndrome (SAPHO), Down syndrome, Crohn’s disease, metabolic syndrome, and diabetes [2,13]. Recently, two studies analyzing ocular comorbidities in patients with HS demonstrated that uveitis, scleritis, and keratitis were positively associated with HS [12,13]. HS has been demonstrated to be an independent risk factor for the development of inflammatory eye diseases (IEDs); in fact, Saygın et al. observed that up to one-third of the patients with HS and IED did not have any other autoimmune or inflammatory comorbidity that could explain their eye involvement [13]. Uveitis was the most prevalent IED, followed by scleritis and peripheral ulcerative keratitis (PUK). The study suggests similarities between HS and Crohn’s disease in terms of impaired Notch signaling and proinflammatory cytokine production and reports a potential association between HS and IED [13].

The eye is a commonly affected organ in several systemic inflammatory diseases including spondylarthritis-related immune diseases, such as psoriasis, psoriatic arthritis, Crohn’s disease, and ulcerative colitis; and vasculitis, such as Behçet’s syndrome, microscopic polyangiitis, and systemic lupus erythematosus [2,12,13]. Understanding the range and patterns of ocular manifestations of systemic inflammatory disease is useful in the diagnosis and management of these patients. Therefore, it is generally recommended to investigate ocular history and symptoms to identify HS patients who should undergo an ophthalmologic examination [12,13].

The retina is a structure that contains a rich network of microvasculature [14]. Digital fundus photography allowed qualitative retinopathy grading, which is based on the identification of hemorrhages, microaneurysms, or focal arteriolar narrowing, and computer-assisted retinal vessel caliber analysis [15]. The most important parameters and metrics are obtained from arteriolar and venular widths from vessels close to the optic disc [15]. As retinopathy reflects established end-organ damage, detecting changes in retinal vessel caliber that precede more conspicuous and clinically relevant damage may allow earlier interventions for high-risk patients [15,16,17]. Optical coherence tomography (OCT) is a non-invasive imaging technology based on low-coherence interferometry. It generates high-resolution, cross-sectional images from backscattered light, enabling clinicians to assess structural changes in different retinal diseases. However, structural OCT cannot be used to monitor vascular changes because of the low contrast between capillaries and retinal tissue. 

OCT angiography (OCTA) is a new, non-invasive, high-resolution imaging technique that carefully analyzes retinal microvasculature networks without the need to inject intravenous fluorescent dye [18,19,20]. OCTA is able to detect the motion of blood capturing the location of blood vessels. Despite its insensitivity to leakage and the relatively small field of view, the development of OCTA has the potential to improve our knowledge of the physiology and pathophysiology of the eye. Unlike fundoscopy, OCTA can detect subtle microvascular abnormalities affecting the retinal layers and choriocapillaris. The analysis of fundoscopy and OCTA can detect retinal vascular changes that are able to predict the incidence of hypertension, diabetes, chronic kidney disease (CKD), and cardiovascular disease (CVD) [12,13,14]. Li et al. examined the retinal microvasculature in a population with Type 2 Diabetes Mellitus (T2DM), observing that the duration of T2DM was independently linked to foveal vascular density and perfusion density, suggesting that T2DM is responsible for chronic and persistent effects on the retinal microvascular system [12]. OCTA examinations have revealed vessel remodeling and chorioretinal thinning associated with hypertension, diabetes, and CKD. Therefore, it has been suggested that the retina could be a “window” into the inflammatory and cardiovascular status of patients [21,22].

Diabetic retinopathy (DR) is associated with systemic vascular complications likely reflecting widespread microvascular disease, and OCTA has become the tool of choice for the early detection of DR and other retinal vascular diseases [15,20,21,22,23,24]. Recent studies have demonstrated that retinal vessel density and retinal perfusion reflect systemic inflammatory responses [22,25,26] and that retinal and choroidal thickness may serve as potential biomarkers of systemic inflammation [27,28]. Kurumoğlu Incekalan et al. observed that children affected by multisystem inflammatory syndrome (MIS-C) showed significant changes during the acute phase including enlarged foveal avascular zone (FAZ), reduced vessel density in superficial and deep retinal plexuses, and choroidal changes [22].

Retinal vascularization and perfusion have been proven to be important parameters for the evaluation of the oxygenation of retinal tissue. These parameters seem to be associated with future retinal tissue functionality. In diabetic patients, the retinal vessel alterations cause a reduction in retinal perfusion slowly damaging retinal cells with the formation of microaneurysms, exudates, and intraretinal microvascular abnormalities. These alterations lead to an earlier reduction in visual function. These parameters have not yet been studied in the context of HS. For this reason, we suppose that an evaluation of retinal microvasculature and perfusion in HS patients should be investigated to predict possible retinal function damage induced by HS disease.

Despite the widespread use of OCTA for eye diseases, little is known about the retinal microvascular features of HS patients. Therefore, this study investigated OCTA-derived retinal microvasculature parameters to understand if HS disease could impact retinal vascular function and morphology.

## 2. Materials and Methods

A retrospective case–control study of HS patients and age- and sex-matched control cohort were carried out at the Dermatology Department in collaboration with the Institute of Ophthalmology of the University of Modena and Reggio Emilia between January 2022 and November 2023. The study was performed in conformity with the ethical guidelines of the Declaration of Helsinki and in compliance with the ethics committee approval (E.C. 444/2021). HS patients’ selection was based on the following criteria: received the first HS diagnosis more than 5 years before enrollment, denied current or past tobacco smoking habit, showed normal glycated hemoglobin (HbA1c) dosage within the last year, and underwent a complete ophthalmic examination for both eyes, including spectral domain (SD)-OCT and OCTA. Patients affected by any type of diabetes, renal, ocular, or cardiovascular diseases, including arterial hypertension, were excluded. The control group included 15 age- and sex-matched non-smoker, healthy volunteers, who consented to OCTA image acquisition. Epidemiologic and clinical information, including past medical history, HS treatments, and disease stage according to Hurley classification and International Hidradenitis suppurativa Severity Score System (IHS4) were analyzed [1,29]. Disease flares were defined according to a recent consensus definition as HS manifestations that were “new or worsening” [30]. 

**Figure 1:**
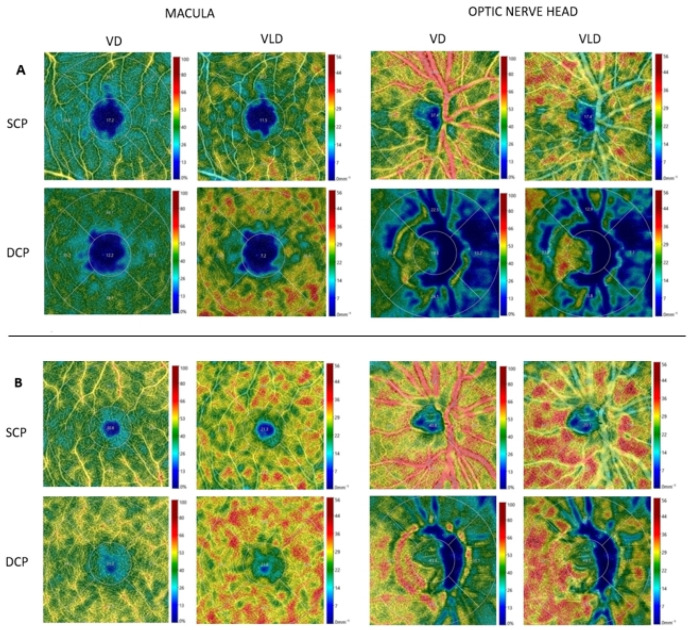
Optical coherence tomography angiography 3 × 3 mm images of retinal perfusion in the macular area and in the optic nerve head area in an HS patient ((**A**), patient n.10) and in a healthy matched control ((**B**), control n.7). A reduction in vasculature texture of both SCP and DCP can be observed in the HS patient. (SCP, superficial capillary plexus; DCP, deep capillary plexus; VD, vessel density; VLD, Vessel Length density).

OCTA acquisition consisted of 3 × 3 mm scans of the superficial capillary plexus (SCP) and deep capillary plexus (DCP) both in the macula and in the optic nerve head, which were performed using Canon OCT HS100 (Canon New Zealand Ltd.) through dilated pupils. The OCTA images of the SCP and DCP were binarized and then skeletonized by an integrated image-processing system software. Vessel density (VD), calculated on a binarized image, was considered as the percentage (%) of white pixels in the region. On skeletonized images, the lines of a binary image were transformed into thin lines (1 pixel of thickness), and the value was obtained by dividing the sum of the length of the thin lines in the region by the area by “mm^−1^” to represent the vessel length density (VLD). All the above-mentioned image processing and segmentation for the retinal vascular plexuses were performed by dedicated software (OCT-HS100 Angio Expert AX^®^, RX Capture for OCT-HS100^®^, Canon, Tokyo, Japan) (Figure 1).

Two ophthalmology specialists, experts in retinal diseases (T.V. and A.L.), performed a detailed review of the OCTA images. VD and VLD were automatically calculated in a fovea-centered circle area (1 mm diameter around the foveola) and in a parafoveal area (an annular area extending between 1 and 2.5 mm diameter, centered on the foveola) segmented into four quadrants (superior, nasal, inferior, and temporal), and then density parameters were recorded for each of them. Furthermore, the mean values of VD and VLD in the foveal area, in the parafoveal area, and in the whole macular area were recorded in SCP and DCP for each patient. Finally, the foveal avascular zone (FAZ) was automatically calculated by the OCT-integrated software for both SCP and DCP. 

## 3. Statistical Analysis

Absolute and relative frequencies of qualitative variables and mean ± standard deviation (SD) for continuous measurements were calculated for each parameter. The Student *t*-test and Fisher exact test were calculated to compare HS and control groups continuous variables (SPSS v. 17 Inc., Chicago, IL, USA). Bonferroni was used (critical α = 0.05) to control Type I error for multiple comparisons [31]. A *p*-value less than 0.0028 was considered significant.

## 4. Results

A total of 20 eyes from 10 HS patients and 30 eyes from 15 healthy controls were included in this study. HS patients’ mean age was 38 years (range 23–57) and the mean BMI was 29.2 (range 20.3–42.5). Time from disease onset varied from 5 to 21 years (average: 9) before enrollment, with pediatric-onset being reported in two patients (20%). Most HS patients were female (60%) and all of them had a history of multiple HS medical or surgical treatments, with subsequent recurrence (average: 2.9, range: 2–5). The disease severity, according to IHS4, ranged from 2 to 34 (mean 10.2). According to Hurley’s classification, 30% of patients were classified as stage I, 40% as stage II, and 30% as stage III. Disease flares were reported with an average of 12.6 episodes every 12 months (range 6–30) (Table 1).

OCTA images showed that, compared to controls, HS patients were typically characterized by significantly higher values of FAZ both in the SCP and in the DCP, increased optical nerve VD in the DCP, and lower values of foveal VD-SCP, VD-DCP, and VLD-SCP (Table 2). Of note, the average foveal VLD-DCP values were lower in HS patients compared to controls, but the *p*-value did not meet the cutoff (*p* < 0.0028) after applying the Bonferroni correction.

## 5. Discussion

In this study, we examined HS-affected individuals with no ophthalmoscopic alterations using OCTA to assess whether retinal microvascular abnormalities occur more frequently in patients as compared to healthy controls. The potential association between HS and several inflammatory eye diseases might be a manifestation of a common immune dysregulation phenomenon that ultimately could affect the microvascular compartments both of the eye and skin. In the skin, it was recently demonstrated that the vascular analysis of actively inflamed HS nodules with a cutaneous D-OCT device could give relevant information regarding patient endotype and risk of disease progression, with possible clinical consequences for therapeutic management and treatment efficacy monitoring [32]. 

According to our preliminary data, we detected several retinal microvascular changes in HS patients. We were able to identify the following: (i) reduced foveal VD and VLD in the SCP and reduced foveal VD in the DCP, (ii) an increased FAZ both in the SCP and DCP, and (iii) increased nerve VD in the DCP. These observations partially reflect the changes that have been demonstrated in diabetic patients, with or without DR [20,33]. OCTA not only allows for the quantification of microvascular changes within the retinal capillary network but also aids in identifying classical DR features such as microaneurysms, intraretinal microvascular abnormalities, and neovascularization. The ability of OCTA to assess the degree of microvascular damage and to identify the eyes with visually impairing macular ischemia in diabetic patients makes it a potentially useful objective approach. Additionally, OCTA could play a significant role in detecting early-stage microvascular abnormalities that precede clinically detectable DR onset [34].

In diabetic patients, the reduction in parafoveal VD-SCP has been previously described as a predicting biomarker for visual impairment [35]. In addition, a significant FAZ increase has been described in several diabetic cohorts [23,33], further highlighting the association between neuronal degeneration and microvascular changes in diabetes mellitus [36,37].

The correlation between HS and type II DM has already been proven by several large epidemiological studies [38], but the inflammatory changes occurring in the retinal vasculature of HS patients in the absence of diabetes have not yet been described. Recent large-scale proteomic studies may indicate a biological explanation of the inflammatory changes observed in OCTA of HS patients [9]. The dysregulation of several molecules implicated in the Th1/Th17 response, together with the upregulation of atherosclerosis-related mechanisms, could be relevant in the generation of microvascular retinal changes in HS patients. It is intriguing to observe that the same immune mediators, such as CD40 ligand, G-CSF, IL-6, and IL-8, are hyper-expressed both in the serum of HS patients and in the aqueous humor of DR patients [9,39]. In addition, the downregulation of CD200 and IL-34, which act as immunoregulatory molecules in the serum of HS patients, could negatively influence microglia-mediated retinopathy, as has been recently demonstrated in the diabetic murine model [40]. Therefore, aberrant inflammatory mechanisms and microglia dysregulation may be the leading pathogenic mechanisms inducing significant OCTA changes both in diabetes and HS.

Recently, OCTA has demonstrated its usefulness in the evaluation of several inflammatory vascular conditions, particularly those that impact the choroid and retina. De Carlo et al. proved that eyes with birdshot retinochoroiditis had aberrant telangiectatic arteries as well as an increase in intercapillary gaps, and they described capillary loops and dilations in most of the analyzed eyes [41]. In the aforementioned diseases, it has also been reported that there was a decrease in VDs in the SCP and DCP as identified by OCTA [42]. According to Spaide et al., OCTA scans in cases of ocular toxoplasmosis revealed that the retinal, and not choroidal vasculature, made a major contribution to the neovascular tissue. Therefore, OCTA can be used to evaluate the origin of aberrant arteries arising in other inflammatory diseases also [43]. In line with Yilmaz Tuğan et al., the Multisystem Inflammatory Syndrome in Children (MIS-C) was associated with lower VD in the SCP as compared to healthy controls, together with a reduced flow in the choriocapillaris layer and outer retina [22]. 

This pilot study shows a reduction in retinal vasculature perfusion parameters in HS patients demonstrating that a retinal OCTA evaluation could be a useful tool to monitor the status of microvasculature in such patients. The therapeutical strategy in HS patients could be improved by oral supplementation of antioxidants and immunomodulatory drugs to control the systemic disease and slow the progression of retinal microvasculature damage. Early treatment has been associated with better outcomes in several inflammatory diseases, including inflammatory bowel diseases and ankylosing spondylitis, reducing the risk of developing bowel strictures and irreversible bone damage leading to new bone formation, respectively [44]. Regarding HS, it was recently demonstrated that early immunomodulatory intervention is associated with a better clinical outcome, preventing the development of skin lesions, such as fistulas, sinus tracts, and scarring sequelae [44]. According to our findings, it is reasonable to hypothesize that early HS immunomodulatory treatment could prevent the occurrence of irreversible retinal microvascular changes, supporting the HS “window of opportunity” theory [44].

There are several limitations to this study. Firstly, the small sample of patients with HS and the retrospective nature of the study: a greater number of cases would be required with a prospective study design. Secondly, ophthalmic imaging was performed both at different time points in the course of the disease and in patients undergoing different treatments, which possibly constituted significant confounding factors.

In conclusion, the structural and blood flow modifications in retinal microvasculature probably occur years to decades before the onset of clinically relevant retinopathy and may be driven by severe systemic inflammatory changes. In this clinical setting, OCTA enables large-scale non-invasive visual screening and follow-up of the retinal vasculature features, providing a new strategy for the prevention and monitoring of visual changes in HS patients.

## Figures and Tables

**Table 1:** HS patients’ characteristics. Epidemiologic, clinical, and HS disease features of the enrolled HS population are summarized.

HS Patients’ Characteristics		
Age, average years (range)		38.2 (23–57)
Sex	Female: *n* (%)	6 (60.0%)
	Male: *n* (%)	4 (40.0%)
HS family history	No: *n* (%)	8 (80.0%)
	Yes: *n* (%)	2 (20.0%)
Body weight, mean kg (range)		83.8 (50–128)
Height, mean cm (range)		168.7 (157–190)
BMI, mean (range)		29.2 (20.3–42.5)
Time from disease onset, average years (range)		9.1 (5–21)
Pediatric onset, *n* (%)		2 (20.0%)
Axillary (%)	No: *n* (%)	3 (30%)
	Yes: *n* (%)	7 (70%)
Pubic (%)	No: *n* (%)	1 (10%)
	Yes: *n* (%)	9 (9%)
Inguinal (%)	No: *n* (%)	2 (20.0%)
	Yes: *n* (%)	8 (80.0%)
Genitals (%)	No: *n* (%)	7 (70%)
	Yes: *n* (%)	3 (30%)
Gluteal (%)	No: *n* (%)	2 (20.0%)
	Yes: *n* (%)	8 (80.0%)
Atypical sites (%)	No: *n* (%)	7 (70%)
	Yes: *n* (%)	3 (30%)
Pilonidal cyst (%)	No: *n* (%)	8 (80.0%)
	Yes: *n* (%)	2 (20.0%)
Hurley stage	1: *n* (%)	3 (30%)
	2: *n* (%)	4 (40.0%)
	3: *n* (%)	3 (30%)
Number of flares, mean per year (range)		12.6 (6–30)
IHS4, mean (range)		10.2 (2–34)
VAS, mean (range)		5.9 (2.3–10)
Number of previous antibiotics, mean (range)		2.9 (1–5)
Current anti-TNF therapy	No: *n* (%)	6 (60.0%)
	Yes: *n* (%)	4 (40.0%)

Body mass index (BMI); International Hidradenitis Suppurativa Severity Score System (IHS4); visual analog scale for pain (VAS). HS atypical sites included: scalp, retroauricular and preauricular skin, chest, thighs, abdomen, posterior neck, and legs.

**Table 2:** Average OCTA values obtained for HS patients and controls.

			HS	Controls	
			Mean, SD	Mean, SD	*p*-Value
Macula	Foveal	VD SCP	23.950, 3.671	26.960, 2.929	0.002 *
		VD DCP	20.245, 6.178	25.540, 4.804	0.001 *
		VLD SCP	15.485, 2.971	18.040, 2.529	0.002 *
		VLD DCP	13.555, 4.819	17.103, 3.844	0.006
	Perifoveal	VD SCP	39.786, 3.164	39.042, 2.639	0.372
		VD DCP	41.587, 2.991	41.043, 2.772	0.513
		VLD SCP	25.441, 1.786	25.160, 1.643	0.571
		VLD DCP	28.979, 1.361	29.178, 1.111	0.574
	Whole	VD SCP	36.669, 2.911	36.646, 2.441	0.976
		VD DCP	37.263, 3.429	37.919, 2.949	0.474
		VLD SCP	23.450, 1.712	23.670, 1.645	0.651
		VLD DCP	25.875, 1.983	26.686, 1.666	0.125
FAZ SCP			0.277, 0.074	0.208, 0.065	0.001 *
FAZ DCP			0.419, 0.108	0.311, 0.091	0.000 *
Optical Nerve	Whole	VD SCP	50.519, 4.494	51.452, 4.627	0.483
		VD DCP	35.387, 2.569	30.601, 6.341	0.002 *
		VLD SCP	25.533, 1.886	26.337, 1.946	0.154
		VLD DCP	20.384, 2.107	18.816, 4.021	0.116

Vessel density % (VD); vessel length density mm^−1^ (VLD); superficial capillary plexus (SCP); deep capillary plexus (DCP); foveal avascular zone mm2 (FAZ). * *p*-value is less than 0.0028.

## Data Availability

Data is unavailable due to privacy restrictions.
